# The experience of hope in dyads living with advanced chronic illness in Portugal: a longitudinal mixed-methods study

**DOI:** 10.1186/s12904-024-01528-x

**Published:** 2024-08-14

**Authors:** Filipa Baptista Peixoto Befecadu, Maria Gonçalves, Cláudia Fernandes, Carlos Laranjeira, Maria dos Anjos Dixe, Ana Querido, Sophie Pautex, Philip J. Larkin, Gora Da Rocha Rodrigues

**Affiliations:** 1 Palliative and Supportive Care Service, Chaire Kristian Gerhard Jebsen of Palliative Care Nursing, Lausanne University Hospital and University of Lausanne, Lausanne, Switzerland; 2 Lausanne University Hospital, Lausanne, CHUV Switzerland; 3 Institute of Higher Education and Research in Healthcare (IUFRS), University of Lausanne, Lausanne, Switzerland; 4 Geneva University Hospitals (HUG), Geneva, Switzerland; 5 Palliative Care Department, Hospital da Senhora da Oliveira, Creixomil Guimarães, Portugal; 6 School of Health Sciences, Polytechnic University of Leiria, Leiria, Portugal; 7 Center for Innovative Care and Health Technology (ciTechCare), Polytechnic University of Leiria, Leiria, Portugal; 8 Comprehensive Health Research Centre (CHRC), University of Évora, Évora, Portugal; 9 Center for Health Technology and Services Research (CINTESIS), NursID, University of Porto, Porto, Portugal; 10 Department of Readaptation and Geriatrics, Palliative Medicine Division, University Hospital Geneva and University of Geneva, Geneva, Switzerland; 11 Geneva School of Health Sciences, HES-SO University of Applied Sciences and Arts Western Switzerland, Geneva, Switzerland; 12 HESAV School of Health Sciences, HES-SO University of Applied Sciences and Arts Western, Lausanne, Switzerland

**Keywords:** Hope, Dyad, Chronic illness, Palliative care, Mixed methods, End-of-life

## Abstract

**Background:**

Hope is an important resource that helps patients and families thrive during difficult times. Although several studies have highlighted the importance of hope in different contexts, its specific manifestations in the realm of advanced chronic illness need further exploration. In this study, we sought to elucidate the intricate interplay between the construct of hope and the lived experience of advanced chronic illness within patient-caregiver dyads. Our objectives were (a) to explore the dyadic experience of hope as a changing dynamic over time for patients living with advanced chronic illness and their informal caregivers and (b) to evaluate variations of hope and symptom burden across time.

**Methods:**

We conducted a longitudinal mixed-methods study with a convergent design between December 2020 and April 2021. Patients living with advanced chronic illness and informal caregivers participated as a dyad (*n* = 8). The Herth Hope Index scale was used to measure dyads' level of hope and the Edmonton Symptom Assessment System was used to measure patients’ symptom burden. Descriptive statistics were undertaken. A thematic analysis as described by Braun and Clarke was conducted to analyze dyadic interview data. Dyads' experience of hope was described by using the six dimensions of hope in the Model of Hope of Dufault and Martocchio.

**Results:**

Dyadic scores of hope and patients' symptom burden were stable over time. The constructs of hope in dyads included “Living one day at the time,” “Having inner force/strength,” and “Maintaining good health.” Changes in patterns of hope were captured for each dyad in their transition over time. Data converged for all dyads except one.

**Conclusions:**

The findings of our study show a constant presence of hope even in the face of adversity. Healthcare professionals must find ways to promote hope in dyads of patients living with advanced chronic diseases. Nurses play a pivotal role; dyadic interviews should be promoted to create a safe space for both patients and informal caregivers in order to share experiences. More research is needed to address patients' and informal caregivers' hope in chronic illness because current hope-based interventions primarily target cancer diagnoses.

**Supplementary Information:**

The online version contains supplementary material available at 10.1186/s12904-024-01528-x.

## Background

Chronic diseases are a major burden on healthcare systems worldwide and are responsible for 41 million deaths each year [1, 2]. The World Health Organization defined chronic diseases as conditions that tend to be of long duration and are the result of a combination of genetic, physiological, environmental, and behavioral factors [1]. The main types of chronic diseases are cardiovascular diseases, cancers, chronic respiratory diseases, and diabetes [1]. As these diseases evolve, they transition into advanced stages in which symptoms become more severe, complications arise, and the impact on an individual’s quality of life intensifies [2]. Effective management and timely interventions are crucial to mitigate the consequences of advanced chronic diseases [2].

Hope emerges as a beacon of resilience in the midst of this challenging journey of living with advanced chronic illness [3]. Hope is defined by Dufault and Martocchio [4] as “ a multidimensional dynamic life force characterized by a confident yet uncertain expectation of achieving a future good which, to the hoping person, is realistically possible and personally significant” (p. 380). This concept has been the object of academic study in both the arts and sciences [5], but a comprehensive definition is elusive. Evidence shows that hope is an important resource that positively influences quality of life and contributes to the comfort and well-being of patients and families [6, 7]. In contrast, feelings of hopelessness are associated with symptom burden, including pain, anxiety, depression, fatigue, spiritual distress, and thoughts of suicide [6, 8, 9]. When patients and families face the consequences of chronic advanced illness, they may need to reassess what hope represents to them, especially when hope for a cure is unlikely [10]. Yet, hope remains present even if life is threatened by near death or if no cure is possible [5].

A recent systematic review and narrative synthesis that explored patients' perception of hope in palliative care highlights the significance of recognizing patients' comprehension of hope, its function, and the endeavors necessary to maintain it toward end of life [11]. The authors indicate that hope functions as a valuable approach, nurturing meaningful interpersonal connections [11]. Furthermore, they suggest that healthcare professionals can address communication challenges of clinical practice by orchestrating hope-based interventions that involve the participation of patients' family and friends [11]. Although hope has been described as a positive resource that enables patients to cope with challenging situations in the context of advanced chronic diseases, hope can also be seen as an obstacle or a barrier when patients deny or avoid their situation or when expectations are unrealistic [12]. In a qualitative study that aimed to examine the meaning of hope of 12 patients with amyotrophic lateral sclerosis, findings revealed that a number of those patients perceived hope as a barrier to empowerment, especially when acceptance of the reality of the progressiveness of the disease was not achieved [12]. The value of hope and how hope acts as a resource to empower and help patients cope and increase their spiritual well-being is widely described in the literature [12, 13]. Even though hope has been widely explored, the focus has mostly been directed toward cancer patients [14–17] or cancer caregivers [18, 19], with limited scope for translation to a chronic illness population.

As patients reach advanced stages of chronic diseases, their symptom burden increases, and functional decline becomes more pronounced [20]. Over time, patients tend to become more dependent on informal caregivers [20]. An informal caregiver is an individual who consistently dedicates their time, typically at least once a week, to support a family member or a loved one of any age who is facing an illness or a decrease in their ability to be independent [21]. This caregiver could be a spouse, parent, child, sibling, friend, or neighbor. Generally, the informal caregiver has no professional training, no working contract, and no monetary compensation [21]. Informal caregivers provide complex care for their loved ones [20, 22] and experience feelings of fear, uncertainty, loss, separation, and the trauma of imminent death when caring for and supporting patients with advanced chronic illness [23–25]. Often, they are overwhelmed, at risk of poor physical and psychological outcomes, surpass their own personal limits, and become exhausted and susceptible to illness themselves [24, 25]. Hope as a coping mechanism becomes vital for both patients and informal caregivers [18]. A systematic review of 26 studies that examined factors associated with hope in family caregivers of persons living with chronic illness suggests a possible link between hope in informal caregivers and their overall positive health [22]. Indeed, the authors found that, as hope levels increase, informal caregivers’ coping abilities improved, including self-efficacy and preparedness for caregiving tasks, whereas maladaptive coping strategies decreased the levels of hope [22]. In addition, feelings of anxiety, depression, distress, grief, and guilt were negatively associated with the level of hope experienced by informal caregivers [22]. Sociodemographic characteristics of informal caregivers seem to be not related to hope [22].

The dynamic interplay between patients and their informal caregivers not only influences individual experiences, but also significantly shapes coping strategies, decision-making processes, and overall well-being within the context of advanced chronic illness [26]. This intricate relationship shows the fundamental role of dyadic interactions in navigating the complexities of advanced chronic diseases. A dyad is defined as an interactive relationship between two individuals [27]. Patients and informal caregivers are usually considered separately, and models of care have therefore tended to target one or the other group, but rarely both together [28]. As a result, these models have limited focus and fail to capture the factors that inhibit or enhance the reality of care [28]. Furthermore, the way in which patients and informal caregivers co-produce their experience of illness is hardly explored in the literature [22, 29]. A cross-sectional study of dyads of hemodialysis patients and their family caregivers showed that hope scores for each person were indicative of their own higher quality of life scores [30]. Hope was measured with the Herth Hope Index (HHI) and quality of life with the World Health Organization Quality of Life-BREF (WHOQOL-BREF) instrument [30]. Patients’ hope scores were associated with improved quality of life in the environmental domain for family caregivers [30]. Thus, to improve quality of life, clinicians should target not only the patient, but also their informal caregivers because of the reciprocal influence. However, few studies have focused on dyads’ experience of hope when dealing with chronic illness [7, 26, 30–32], in particular for patients with non-oncological illnesses [18]. As part of a multicenter longitudinal mixed-methods study of dyads of patients with chronic obstructive pulmonary disease and their informal caregivers, this exploratory study provided a valuable foundation for refining the methodology in our current study [26]. Working at a dyadic level enables a relationship-based understanding of dyadic behaviors and coping strategies to emerge, thus managing illness that affects the health and well-being of both patients and informal caregivers [28].

Palliative care aims to enhance the quality of life for patients and families who are facing life-limiting illnesses by addressing physical, psychosocial, and spiritual needs through early identification, accurate assessment, and treatment to prevent and alleviate suffering [33]. Palliative care must emphasize healthy transitions, which generates opportunities for healthcare professionals to assist dyads according to their needs [34]. Such an approach aligns with Meleis’ [34] theory by recognizing the interconnectedness between the patient and their informal caregiver, emphasizing the need to offer tailored support to both individuals within the dyad. This support can include personalized care plans, psychological support, education, and resources aimed at addressing the unique challenges and requirements of the dyadic relationship, ultimately contributing to a more effective and holistic palliative care experience [34]. Nurses play a pivotal role in palliative care, providing comprehensive support and coordination of care for patients and their families. As healthcare professionals, nurses have a key role in the facilitation of healthy transitions for dyads in accordance with their individual needs [34]. Moreover, their expertise, compassion, and holistic approach to care can profoundly influence the hope experience of dyads [35].

Our aims in this exploratory study were (a) to explore the dyadic experience of hope as a changing dynamic over time for patients living with advanced chronic illnesses and their informal caregivers and (b) to evaluate variations of hope and symptom burden across time in dyads. We hope that this study provides an expanded and more comprehensive understanding of the multidimensional phenomena of hope in dyads.

## Methods

Using a convergent design, we conducted a longitudinal mixed-methods study [36]. To gain a deeper understanding of transitional patterns of hope focused on dyadic experience over time, we collected data through semi-structured dyadic interviews and a survey at two time points (T1: baseline, T2: 4 months after baseline). The merits of this method lie in its capacity to flexibly assess the interplay between time and context in a nonlinear manner [37, 38].

This study was conducted between December 2020 and April 2021 in a 485-bed public hospital in the north of Portugal, and it adheres to the recommendations for Good Reporting of a Mixed Methods Study (GRAMMS) [39].

### Setting and sample recruitment

The study took place in the palliative care outpatient clinic of a public hospital in Portugal. The population consisted of patients with non-cancer life-limiting illness (advanced/incurable chronic illness) and/or limited life expectancy (according to Prognostic Indicator Guidance from the Gold Standards Framework [40]) and their informal caregivers. The informal caregivers were identified by the patient as the person most involved in care and support (emotional or physical support). We based the inclusion criteria on the specific clinical indicators related to organ failure trajectories described in the Gold Standards Framework, and we included the surprise question as a means of identifying patients whose state of illness was advanced and who may therefore have benefited from palliative care [40]. Participants’ inclusion and exclusion criteria are presented in Table 1.

**Table 1:** Eligibility criteria of participants

Inclusion criteria	Exclusion criteria
1. Patient or informal caregiver sufficiently literate in Portuguese language to understand written information or be able to understand and engage with the interview questions without difficulty 2. Adult patient diagnosed with non-cancer conditions [40]: - severe chronic lung diseases or - chronic heart failure or - chronic kidney disease or - stroke with severe functional impairment or - chronic liver disease or - motor neuron disease with marked rapid decline in physical status or - multiple sclerosis with major complex symptoms or - HIV/AIDS 3. Patient whose general health status is suitable for participation in the study based on a clinical judgement of the clinical team 4. Clinician advice based on the negative answer to the “surprise question”: “Would you be surprised if this patient died in the next 12 months?” [41]	1. Patient with an anticipated prognosis of less than 3 months 2. Patient diagnosed with active cancer, receiving curative or palliative cancer therapies 3. Cognitive impairments screened by using the Portuguese version of the Short Portable Mental Status Questionnaire (≥ 6 errors) [42]

Participants were purposively selected by the hospital-based palliative care consultation team and research team together. This method was chosen to enhance the quality, relevance, and depth of the data collected, ensuring that our study could provide meaningful and accurate insights [43]. Upon identification, potential participants were approached either during their clinic visits or through direct communication facilitated by their healthcare providers. Nine potential dyads were identified and approached to participate in the study. Among these dyads, eight agreed to participate and one declined. The reason for refusal was a personal reason unrelated to the study’s objectives, mostly related to the time constraints of the informal caregiver.

### Data collection

Data collection was undertaken by two registered nurses: FBPB, a doctoral student and clinical nurse specialist in palliative care with a master’s in nursing sciences, and CF, a clinical nurse specialist in palliative care with a master’s degree in palliative care, both trained in qualitative interviewing. Neither had prior contact with the patients or the informal caregivers. CF, working as part of the hospital-based palliative care consultation team, was responsible for identifying eligible patients with MG, the head-physician of the palliative care team. When eligible participants were identified, researchers would present the study to them and give them at least 24 h to decide if they wanted to participate or not. After receiving informed consent from the participants, the research team called them and set an appointment at the palliative care outpatient clinic, where T1 data collection would take place. Participants were given the choice of the place of the interview and data collection; all preferred to meet the research team at the hospital.

### Instruments

Quantitative data collection involved the following three parts.

1. Sociodemographic and clinical data from the patient’s chart. These data included the following variables: age (years), marital status (single/married/divorced), education (1st, 2nd, 3rd cycles, secondary and higher education), professional situation (employed, retired, sick leave), religious beliefs, medical diagnosis, and date of diagnosis (years).
2. Portuguese version of the Herth Hope Index (HHI) [44–46]. The HHI is a reliable instrument to measure the level of hope in patients with life-limiting illnesses and their informal caregivers. The HHI is a 12-item, 4-point Likert scale with a possible score ranging between 12 and 48 and higher scores indicating higher levels of hope [45, 46]. Given that the scale performs differently among patients and caregivers, we opted to use the original structure composed of 12 items distributed among three factors [44]. Factor 1 is related to the person’s inner sense of temporality and future (items 1, 2, 6, and 11) (range: 4–16), Factor 2 concerns one’s inner positive readiness and expectancy (items 4, 7, 10, and 12) (range: 4–16). Factor 3 is about the interconnectedness with self and others (items 3, 5, 8, and 9) (range: 4–16). The Cronbach’s alpha of the original version of HHI was 0.97 [44]. Two items of this scale are reversed (items 3 and 6) to counteract response bias [44].
3. Edmonton Symptom Assessment System (ESAS) [47, 48]. The ESAS is a nine-item scale of patient-rated symptoms (Pain, Fatigue, Nausea, Depression, Anxiety, Drowsiness, Appetite, Well-being, Shortness of breath) with a numerical rating of symptom severity (0–10) [47]. The total scores range from 0 to 90 and higher scores indicate greater symptom burden [47].
  Informal caregivers completed only the HHI. Each participant completed the questionnaires separately in the presence of one of the research members. The reliability of the instrument reveals good indicators.

Qualitative data were obtained by using a semi-structured dyadic interview guide developed by the authors. Dyadic interviews recognize the interdependence between individuals and involve one interviewer and two interviewees interviewed together [49, 50]. The semi-structured interview guide was developed to capture the essence of the dyad experience of hope, inspired by the six-dimension Model of Hope by Dufault and Martocchio [4]. The interview guide was revised and validated by a committee of experts in health-related qualitative method studies (GDRR, AQ, CL, and PL). The content of the finalized semi-structured interview guide is presented in Table 2.

**Table 2:** Semi-structured interview guide based on conceptual dimensions of hope proposed by Dufault and Martocchio [1]

Conceptual base	Question
Definition of the concept of hope	When I speak to you about the word hope, what are the first thoughts that come to your mind?
Contextual	Given the situation at the moment, what makes you a hopeful person? What is the meaning/significance of your life?
Cognitive	If you had to tell someone what it takes to have hope, what would you tell them? Can you identify a source of hope for you? What gives hope or takes away your hope?
Affective	In light of the situation you are experiencing, how do you maintain hope? (emotions/feelings, doubts…)
Affiliative	In what way do others (family, friends, luck, fate, God) influence your hope? How do they help you to keep your hope alive?
Behavioral	What do you hope for in your life (goals/motivations)? Do you have plans? (Tell me about …)
Temporal	In which way has time changed your experience of hope?

### Procedures

Qualitative interview data and quantitative data were collected at the same time at two points (T1: baseline, T2: 4 months after baseline). The 4-month time frame was chosen in order to observe meaningful changes in hope dynamics and symptom burden [51]. Table 3 shows the schedule of the assessments and the interviews.

**Table 3:** Schedule of assessments and interviews

Measures	T1 baseline	T2 (4 months after T1)
Socio-demographic and clinical data	X	
HHI	X	X
ESAS (only for patients)	X	X
Semi-structured interviews	X	X

*HHI* Herth hope index, *ESAS *Edmonton symptom assessment system

Interviewers audiotaped the interviews and took field notes to ensure accuracy and veracity, as well as offering opportunities for reflection during and after the process of data collection. A total of 15 interviews were conducted. The interviews lasted 27 min on average (ranging from 15 to 45 min).

### Data analysis

#### Quantitative

Quantitative data were analyzed by using STATA version 14.2. Descriptive statistics (frequencies, percentages, range, means ± standard deviation) were used to depict the sample over time (T1 and T2).

#### Qualitative

Dyadic semi-structured interviews were transcribed verbatim and analysis was conducted following a codebook thematic analysis approach, as described by Braun and Clarke [52, 53]. Thematic analysis was chosen because of its flexibility and adaptability within a pragmatist paradigm. Furthermore, thematic analysis offers a transparent and systematic process for data analysis, with clear steps for rigorous analysis [52]. From a social constructivist perspective, reality is seen as socially constructed and subjective, and meaning is co-constructed through interactions [54]. This pragmatic alignment allowed us to effectively integrate qualitative and quantitative data, thereby enriching our understanding of the complex, socially constructed nature of hope within the dyad.

Before starting the analysis, recordings of each interview were listened to and notes were taken. A brief description of the context of the interview was also written. The Model of Hope of Dufault and Martocchio and inductive and deductive reasoning were used to interpret the experience of dyads [4]. We used the six dimensions of hope from the Model of Hope as initial codes. The first two interviews were coded by FBPB, GDRR, AQ, and CL separately. The researchers then met to combine analyses and to discuss and agree on codes and themes. After themes and codes were structured, FBPB continued the analysis and met regularly with GDRR, AQ, and CL to validate the analysis. Finally, themes were defined and named, and those definitions were reviewed by all members of the research team. Interviews were analyzed in their original language, and then themes and subthemes were translated into English. WebQDA software was used to analyze and store data.

#### Integration of qualitative and quantitative data

After the questionnaires and interview data were analyzed, the data were merged and reported by using both the Dufault and Martocchio Model of Hope and the middle-range transitions theory of Afaf Meleis to help identify the patterns of transition of dyads living with advanced chronic illness. Herein, we present the dyad’s constructs of hope, which represent the definition of hope of dyads within the context of advanced chronic illness. In addition, we present a descriptive analysis of the dyads’ experience of hope in which the Model of Hope by Dufault and Martocchio served as a blueprint. Finally, we present a description of each interviewed dyad and the changes in their experience over time combined with HHI results, which shows when the data converge or diverge.

Convergence occurs when qualitative and quantitative data corroborate or complement each other, providing a unified understanding or explanation of the research question. For instance, if qualitative interviews reveal a certain trend and quantitative results confirm the same trend, this demonstrates convergence between the two data sets, enhancing the overall validity and reliability of the study. On the other hand, divergence represents inconsistencies between qualitative and quantitative findings. Divergence highlights contrasting results, offering opportunities to explore discrepancies, understand nuanced aspects, or identify limitations within the study. Divergence does not necessarily imply conflict or contradiction; rather, it can offer valuable insights into the complexity of the experience of hope of dyads [55].

### Trustworthiness

To ensure *trustworthiness and credibility,* two members of the team independently analyzed the verbatim transcripts (FBPB and GDRR) and then confirmed the results with other members of the team (PL, AQ, CF, SP) [52]. A codebook based on the six dimensions of hope was developed with additional themes added as they arose from the data and was subsequently validated by the other team members. Comprehensive field notes were taken over time to ensure *transferability* [56]. Longitudinal qualitative data are complex to analyze. As two sets of data were collected at two points in time, the aim was to grasp the changes over time, rather than to obtain a snapshot in time, common to most qualitative studies [57]. *Confirmability* was guaranteed by using triangulation (investigator, methods, and theory), multiple researchers collected data and different theoretical perspectives were used to analyze the data, and different methodologies were used to approach the problem [56]. The thematic analysis, member checking, and presentation of reports were repeated at T1 and T2.

### Human ethics and consent to participate declarations

All procedures were conducted in accordance with the Declaration of Helsinki. The study was granted permission by the ethics committee of the Hospital da Senhora da Oliveira in Guimarães, Portugal (Ref: 86/2020). All dyads included in the study received a participant information letter. Written formal consent was obtained before any data collection.

## Results

### Participants’ backgrounds

A total of eight dyads (16 participants in total) completed baseline measures. Table 4 shows participants’ sociodemographic and clinical characteristics. Most of the dyads were in a close family relationship to the patient, and the age ratio between the patient and the informal caregiver varied depending on the status of that relationship (i.e., married couples tended to be closer in age than parent–child dyads). One patient (in Dyad H), although married, received their main caregiving from friends. Patients presented with a range of advanced chronic diseases conducive to the need for ongoing complex care and support. With one exception, all dyads completed T1 and T2 data collection. The dropout of Dyad C was due to deterioration in health and inability to complete instruments and follow an interview at T2.

**Table 4:** Participants’ sociodemographic and clinical characteristics

Dyad	A	B	C	D	E	F	G	H
Participants (*N* = 8 dyads)	Mother (PT) and son	Wife (PT) and husband	Brothers	Father (PT) and daughter	Mother (PT) and son	Husband (PT) and wife	Husband (PT) and wife	Friends
Patient diagnosis	COPD	ALS	CHF	ILD	Chronic respiratory failure	COPD	Pulmonary fibrosis	ALS
Date of diagnosis	2010	2009	2011	2017	2000	2001	2000	1997
Marital status Patient Informal caregiver	Divorced Single	Married Married	Single Married	Married Married	Married Single	Married Married	Married Married	Married Married
Patient age (58.88 ± 9.46)	58	68	46	61	52	70	48	68
IC age (44.62 ± 19.96)	20	69	43	35	20	74	49	47
Professional situation Patient Informal caregiver	Retired Employed	Retired Retired	Sick leave Employed	Retired Employed	Retired Employed	Retired Retired	Retired Retired	Retired Employed
Religion	Evangelical Protestantism	Catholic	Catholic	Catholic	Catholic	Catholic	Catholic	Catholic
Level of education Patient Informal caregiver	3rd cycle Secondary	1st cycle 1st cycle	1st cycle 3rd cycle	1st cycle Secondary	1st cycle 3rd cycle	1st cycle 1st cycle	3rd cycle 1st cycle	1st cycle 3rd cycle
Number of interviews	2	2	1	2	2	2	2	2

*PT* Patient, *COPD *Chronic obstructive pulmonary disease, *ALS *Amyotrophic lateral sclerosis, *CHF* Chronic heart failure, *ILD* Interstitial lung disease

### Variations of hope over time

**Fig. 1:**
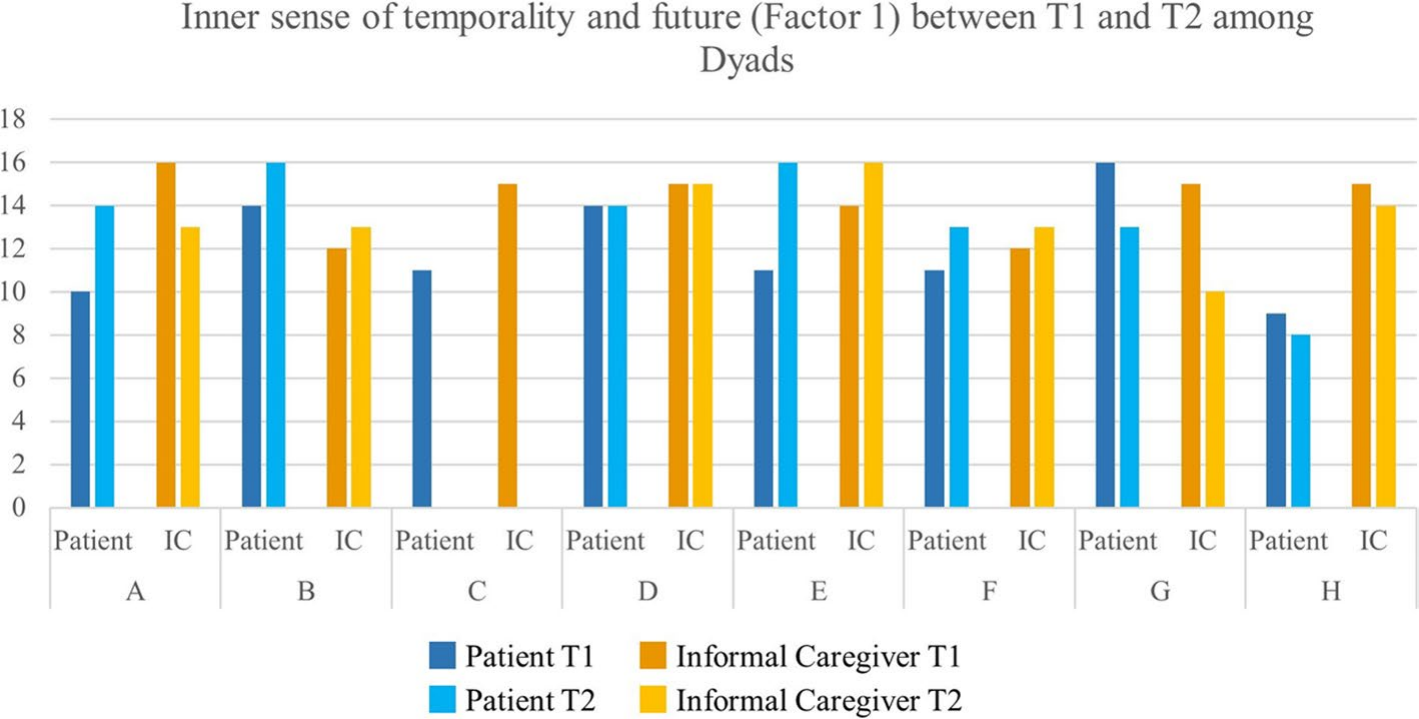
Inner sense of temporality and future (Factor 1) between T1 and T2 among Dyads

**Fig. 2:**
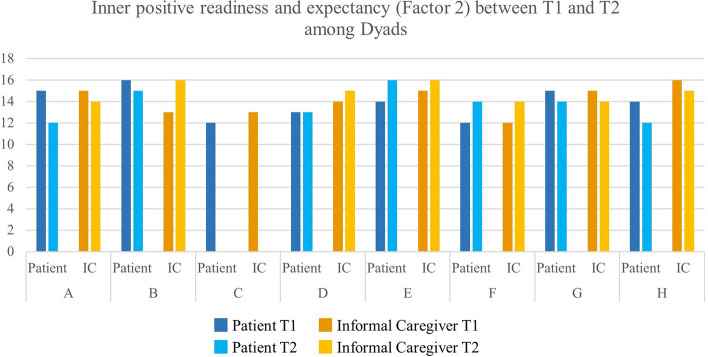
Inner positive readiness and expectancy (Factor 2) between T1 and T2 among Dyads

**Fig. 3:**
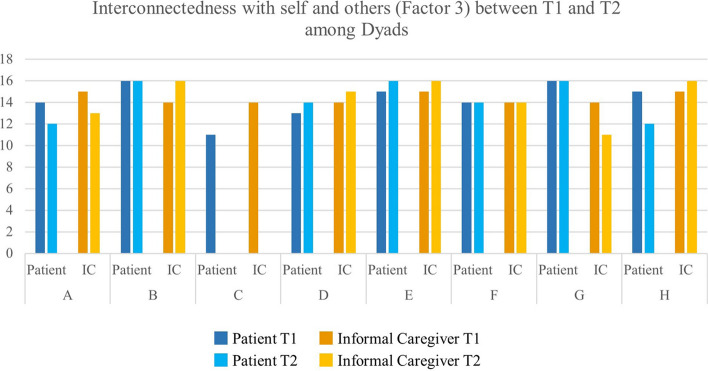
Interconnectedness with self and others (Factor 3) between T1 and T2 among Dyads. Factor 1—Inner sense of temporality and future (items 1, 2, 6, and 11) (range 4–16). Factor 2—Inner positive readiness and expectancy (items 4, 7, 10, and 12) (range 4–16). Factor 3—Interconnectedness with self and others (items 3, 5, 8, and 9) (range 4–16)

Overall, subdimensions of hope were stable across time (see Fig. 1, 2 and 3). However, in some cases, a strong variation between T1 and T2 could be observed. The patient in Dyad A had a relatively low score in Factor 1 at T1, whereas this score increased at T2. Yet, Factor 2 and Factor 3 had high values at T1 and decreased at T2. At the same time, the informal caregiver in Dyad A remained stable between T1 and T2.

### Global scores of hope and symptom burden over time

Participant’s scores of hope were stable over time (see Table 5). Patients had higher scores of hope at T2, whereas informal caregiver results were constant. Symptom burden scores were higher at T2 than at T1.

**Table 5:** Dyads’ HHI and ESAS scores

	**T1**	**T2**	**T1**	**T2**	**T1**	**T2**
Dyad	Patient ESAS score (20.13 ± 12.30)	Patient ESAS score (26.00 ± 15.26)	Patient HHI score (40.13 ± 4.39)	Patient HHI score (41.43 ± 5.44)	Informal caregiver HHI score (42.75 ± 2.96)	Informal caregiver HHI score (42.71 ± 4.35)
A	45	47	39	38	46	40
B	4	6	46	47	39	45
C	18	Dropout	34	Dropout	42	Dropout
D	12	36	40	41	43	45
E	21	12	40	48	44	48
F	23	22	37	41	38	41
G	12	19	47	43	44	35
H	26	40	38	32	46	45

*ESAS* Edmonton Symptom Assessment System, *HHI *Herth Hope Index

Although most patients presented similar ESAS scores at T1 and T2, some (notably patients D and H) showed changes in self-estimated symptom burden between T1 and T2, which was considered unsurprising given the nature of their evolving illness.

### Dyads’ constructs of hope

**Fig. 4:**
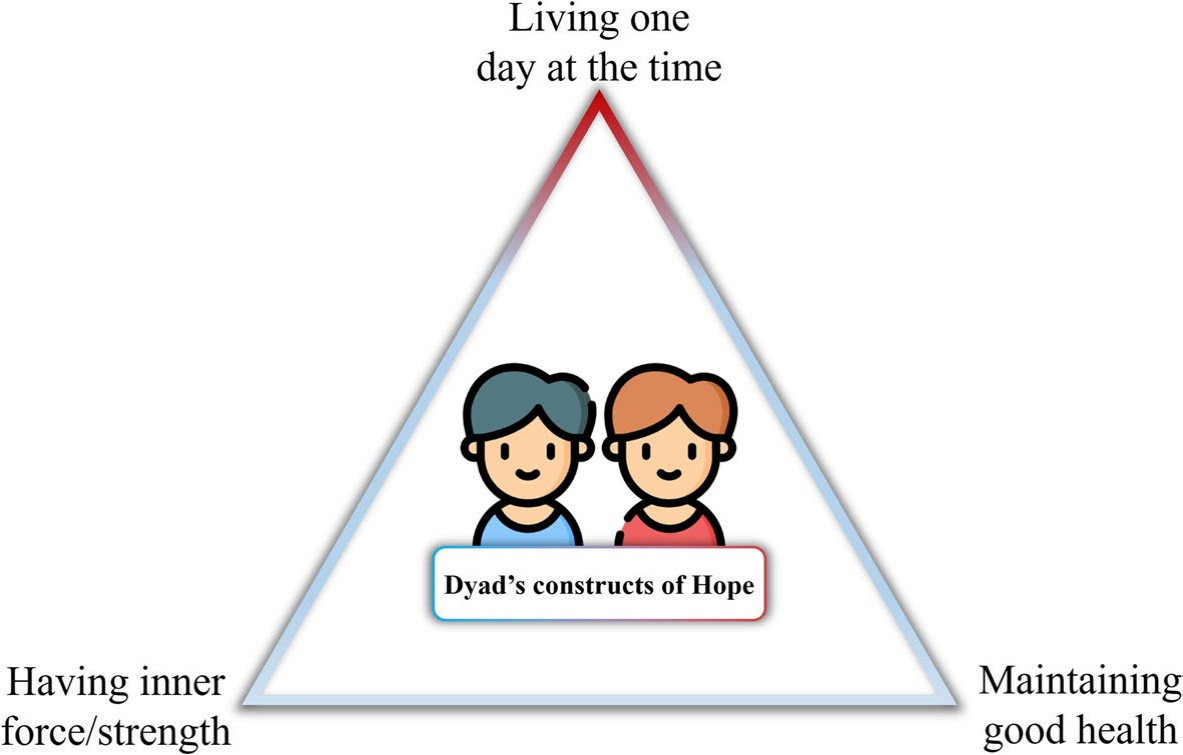
Dyads’ constructs of hope

Dyad’s constructs of hope are presented in the Fig. 4.

From the data analysis, three key themes emerged, offering a comprehensive understanding of the construct of hope for dyads: living one day at a time, having inner strength, and maintaining good health.

#### Living one day at the time

For dyads, the construct of hope is associated with life. To live is having quality of life, having new opportunities, and living one day at the time. For participants, quality of life is having a “normal” life and being healthy to live without restrictions. Dyad A, Patient, T2: “Hope is to have more quality of life, to have quality of life, to be able to live normally, to be able to have a healthy life, to be able to go out, to be able to socialize…”. New opportunities were associated with possibilities, which could include receiving a transplant or having a miraculous treatment. This seemed to be related to how healthcare professionals suggest these treatments.

Dyad G, Patient, T1: “[Hope] It's very simple, it's a [lung] transplant. …. It's the only thing.”

Dyad G, Informal caregiver, T1: “Everything is focused on this situation [the possibility of a transplant]. Then we can go for a walk, hold hands, have coffee, have a romantic date…”.

Living one day at a time was expressed as making no plans and living every day to the fullest possible extent. Dyad D, informal caregiver, T1: “I don't have any plans, because there's no point in making plans; it's better to live day by day intensely and one day at a time, nothing more than that.”

#### Having inner force/strength

Hope is also associated with a force, expressed as a will to live or a force that comes “from inside.” This force is needed to face the evolution of the illness for the dyad. Although sometimes this inner force had to be actively developed to face the consequences of the illness, at other times, it remained elusive. In that case, it was more difficult to activate this personal resource.

Dyad B, Informal caregiver, T1: “But I am the one who has to create strength to face what I have…” Dyad H, Patient, T1: “What keeps me hopeful is to be strong, to make myself strong, and to keep myself in the best way so that my husband doesn't feel sad.”

Dyad H, Informal caregiver, T2: “I think I have a strength that I don't know where it comes from…”.

#### Maintaining good health

Hope also means being healthy; the absence of a healthy state might induce a threat to hope.

Dyad H, Patient, T1*:* “To me what comes to mind…hope…, is health, which is what I lack…”.

Dyad F, Informal caregiver, T1: “Hoping we'll be healthy, living more or less well for me is this.”

### Model of hope: descriptive analysis of dyads’ experience of hope

**Fig. 5:**
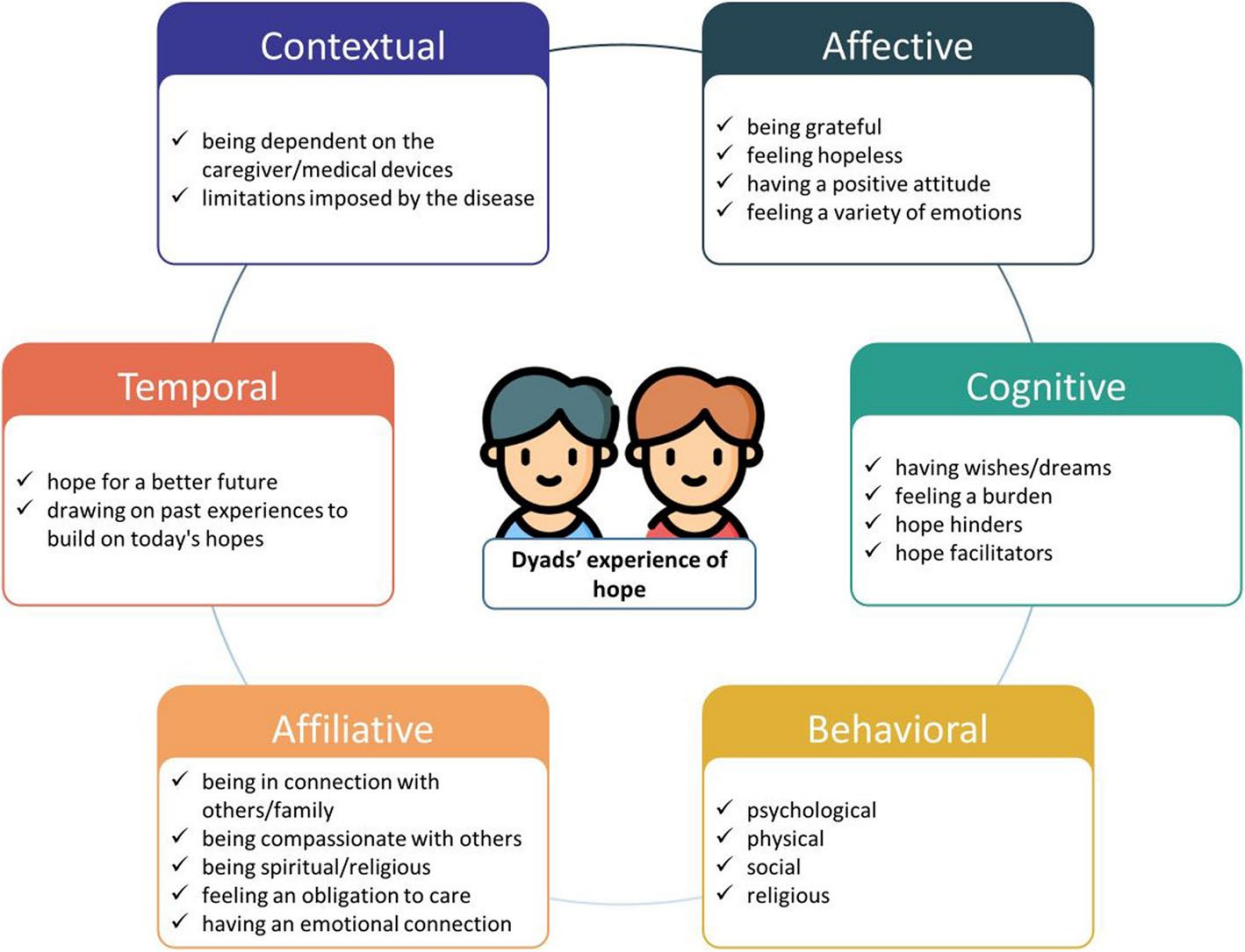
Dyads’ experience of hope

Figure 5 shows a descriptive analysis of the dyads’ experience of hope. The Model of Hope from Dufault and Martocchio [4] was used to describe each dimension.

The *affective dimension* includes sensations and emotions [4]. In our study, we found that dyads tended to feel a large range of emotions over time, including feelings of fear, sadness, happiness, and anxiety. The patient of Dyad E,in her first interview talked about the anxiety of waiting: “Sometimes it's a bit of anxiety, waiting…, sometimes it's difficult. You're always… anxious, always waiting for something to happen [waiting for the transplant].” The patient of Dyad B expressed happiness in small things such as going for a walk or enjoying a meal in a restaurant despite all limitations: “I think like this, I'm happy, I live day to day…I still go for a walk now with my husband, I even go to the restaurant.”

The *cognitive dimension* comprises the wishes and dreams of dyads, the facilitators of and barriers to the maintenance of hope [4]. Dyads’ most common dreams were related to their family and offspring. The patient Dyad G wished to see his daughter going to university: “[Dreams] It's seeing my daughter go to university.” The patient of Dyad E wanted to meet his grandchildren and be there for their children at special events such as their wedding: “The plans I have are to meet my grandchildren and take my children up to the aisle.”

Dyads’ facilitators of hope were expressed through getting good news. The patient in Dyad E was told by her pulmonologist that she meets the criteria for a lung transplant: “When my doctor said that my salvation was the transplant, then everything improved …. It changed my life completely, when the doctor told me, I was very happy…” Factors that hindered dyads’ experience of hope were commonly bad news, which the patient in Dyad H shared: “What takes my hope away? Oh, I don't know… if I get some not so good news.”

The *behavioral dimension* of hope encompasses the action-oriented approach that individuals take toward their desired outcomes, spanning psychological, physical, social, and religious domains [4]. In the psychological subdomain, we found that dyads actively want to hope a lung transplant will save them; for example, the patient in Dyad A expressed the following: “…because I don't know if I'll get it [the transplant] but I have to believe in something.”

In the physical subdomain, keeping active and being in contact with nature were actions taken by dyads that helped to maintain hope. The patient in Dyad E stated: “One thing that I have, that keeps me more in faith, more hopeful, for example, when I have those negative thoughts, I come outside to the garden and spend time with the plants and it's therapy for me.”

In the social subdomain, some dyads reflected on their attitude of isolation toward society or even their loved ones; the brother of the patient in Dyad C expressed sadness over their brother not leaving the house and not putting in effort to lose weight. He emphasized that his brother needs to take responsibility for his own actions and that nobody else can do it for him. Both patient and informal caregiver of Dyad C conveyed a sense of hopelessness. The informal caregiver described his brother as essentially present but not truly living life, likening him to a “dead person standing there.” The Informal caregiver in Dyad C shared the following: “It's sad, it's sad, of course, he doesn't leave the house and he could make a bit of an effort to lose weight. He's the one who has to do it for himself, nobody else can do it, otherwise, he's just a person who's there as if he wasn't there. He's a dead person standing there.” Patient: “It's exactly like that.”

The religious subdomain reflects actions such as prayer and attending Mass, which involves reconnecting with one's faith and spiritual practice. This is represented by the informal caregiver in Dyad F: “I like going to Mass, I like praying, a lot… I pray in the morning. I get up and start praying straightaway.”

The *affiliative dimension* encompasses a sense of connection beyond oneself, involving social interaction, mutual support, attachment, and the acknowledgement that others can significantly impact an individual's hopes [4]. In this dimension, dyads’ experience of hope was expressed as being in connection with others, being compassionate, being spiritual/religious, having an emotional connection, and feeling an obligation to care. Being in connection with others involved receiving support from friends, as illustrated by the informal caregiver in Dyad G: “When you need it most, they're always there, you need something and they're always ready. We live in a village, and there are very few public transport options. If I need something, I call my brother-in-law or a friend; they're always ready.”

Being compassionate with others was part of the experience of hope in Dyad A. The patient spends most of her time at home, as she is not capable of going out often because her building does not have an elevator. However, she is the main support of a friend who needs comfort, and during the interview, she stated: “Every day I send her a message of encouragement. Every day I send her a good night message in the evening, a good morning message to make sure she wakes up better.”

Being religious or spiritual is part of the experience of hope. The words “God,” “hope,” and “faith” frequently coexist in the same sentence. The informal caregiver in Dyad F said: “Have hope! Have faith in God that he will help you, that's what I always tell everyone.”

Emotional connections within the family are an important part of dyads’ experience of hope. During the COVID-19 pandemic, the patient in Dyad D was hospitalized for an extended period without any visits, causing concern about leaving his wife alone at home. His daughter, son-in-law, and grandchildren stepped in to support and stay with his wife, which aided the patient in coping with his own situation, about which the patient said: “…that’s what gave me the most strength… my wife was alone and they [daughter, son-in-law, and grandchildren] went there. It helped me stabilize 50%.”

The informal caregiver in Dyad F conveyed a sense of obligation to look after his mother while his father was not present: “I have a property of my own, but when my father fell ill, I didn't want to leave my mum on her own. I moved in with my mum because it didn't make sense… so I left my house.”

The *temporal dimension* of hope involves the individual's perception of time (past, present, and future) in connection with their hopes and experiences [4]. Dyads’ experience of hope involved hope for a better future and leveraging on past experiences to build on today’s hopes. Hoping things will get better is part of the everyday life of dyads living with chronic advanced illnesses. Informal caregiver of Dyad A illustrates this: “Hope is having a future…. that things will get better in the future.” Past experiences were considered the foundation to nurture present hopes, as reflected on by the informal caregiver in Dyad H: “It's just that as you live you learn more things, you get a different mindset, don't you? Today we are having a bad time, tomorrow we know how to deal with the situation, and I think that ends up giving us another way of looking at life, and at least I have more hope … not everything is good and so we have to deal with things.”

Finally, the *contextual dimension* comprises the life circumstances that shape and constitute an individual’s hope [4]. In our dyads, this was represented as being dependent on the caregiver or on medical devices. The patient in Dyad E explained at which point she was reliant on oxygen in her everyday life: “I have to have oxygen to tidy up the whole house.” The informal caregiver in Dyad G was the wife of the patient and was the main caregiver of her husband. She expressed how she was afraid of bathing him: “The bath … of course I had to do almost everything myself, my biggest fear was that he'd get into the bathtub, and I wouldn't be able to get him out…”.

### Transition between T1 and T2

Two interviews were conducted at T1 and T2. The same dyads were interviewed by using the same interview guide. Dyads’ characteristics and changes over time are presented in Table 6.

**Table 6:** Description of dyads and changes in their experience over time with HHI scores

Dyad	Context	T1 Patient and Informal caregiver level of hope in each factor of the HHI			T1 1^st^ interview	T1-T2 Patient and Informal caregiver HHI overall score		T2 Patient and Informal caregiver level of hope in each factor of the HHI			T2 2^nd^ interview	Mixed-methods Meta-inferences
A	A lot of complicity between mother and son. The mother is a patient who has COPD and is waiting for a lung transplant. She has many limitations in her daily life, being dependent on O_2_ 24 h/day. They are both evangelicals and practice religion actively through praying and reading the Bible.		PT	IC	Hope is focused on lung transplantation and the possibility of living a few more years with QoL. The patient reports that the disease affects her self-esteem in terms of her image. The son talks a lot about his “duty” to take care of his mother. The mother in turn does not want to be a “burden” for her son.	PT	39-38		PT	IC	Hope remains focused on lung transplantation. The objective of the patient is to be able to live longer with QoL and to be with her son. In this interview, the patient introduces the notion of having the feeling that her life is on “stand-by” until she gets the lung.	Congruent The dyad’s hope is described in the same way in both periods and is confirmed by HHI scores that are stable.
		Factor 1 Temporal awareness	10	16		IC	46-40	Factor 1 Temporal awareness	14	13		
		Factor 2 Optimistic readiness	15	15				Factor 2 Optimistic readiness	12	14		
		Factor 3 Interconnectedness	14	15				Factor 3 Interconnectedness	12	13		
B	A couple in which the woman is the patient (ALS) and her husband is the carer. The patient has limitations in activities of daily living, is wheelchair-bound, and needs help with almost everything. She has difficulty articulating words. The husband is the main carer, who manages all of the logistics surrounding his wife's illness.		PT	IC	The patient feels like a happy woman and hopes to live as long as possible. The husband expresses that at the beginning of the discovery of the disease, it was difficult for him, but as time went by, he got used to it and says he tries to do his “best” every day. Outdoor activities, such as going out and traveling, or being in contact with nature, are important parts of this couple's well-being.	PT	46-47		PT	IC	The couple talked again about the moment when they found out about the disease and how difficult it was for them. They also talked about how important it is for them to get out of the house, go for walks, and do activities outside the home.	Congruent The dyad describes their experience as focusing on the positive aspects, which are reflected in the HHI scores.
		Factor 1 Temporal awareness	14	12		IC	39-45	Factor 1 Temporal awareness	16	13		
		Factor 2 Optimistic readiness	16	13				Factor 2 Optimistic readiness	15	16		
		Factor 3 Interconnectedness	16	14				Factor 3 Interconnectedness	16	16		
C	This dyad is composed of two brothers; the younger one is the patient and the older one is the carer. The patient has heart failure and obesity. The interview was very poor in content.		PT	IC	The patient focuses on his experience of wanting to be healthy so that he can live and work. Sometimes the speech is contradictory and difficult to understand. The carer has not shared much about his experience. He regrets his brother’s situation, saying that it is a “sad” situation of social isolation (his brother does not go out).	_					_	-
		Factor 1 Temporal awareness	11	15								
		Factor 2 Optimistic readiness	12	13								
		Factor 3 Interconnectedness	11	14								
D	Father and daughter, in which the patient is the father who has pulmonary fibrosis and was a victim of COVID in the first wave. He was offered the possibility of a transplant, but refused.		PT	IC	Patient with great faith in God: “those who have faith have hope.” The patient talks about his experience as a COVID patient hospitalized and away from all of his family. The daughter has a positive attitude toward life and promotes this attitude with everyone around her.	PT	40-41		PT	IC	In the second interview, the experience of COVID was less present and the interview was less deep than the first one. Each time hope is mentioned, the connection with faith and God is very strong, especially for the patient. The daughter continues to relate how her positive attitude toward life helps to maintain hope.	Congruent The dyad describes their experience by concentrating on positive attitudes to face life. These findings are confirmed by the HHI scores.
		Factor 1 Temporal awareness	14	15		IC	43-45	Factor 1 Temporal awareness	14	15		
		Factor 2 Optimistic readiness	13	14				Factor 2 Optimistic readiness	13	15		
		Factor 3 Interconnectedness	13	14				Factor 3 Interconnectedness	14	15		
E	Mother and son, in which the mother is the patient and the youngest son is the carer. The patient has chronic respiratory insufficiency. She needs O_2_ 24 h/day and is on the waiting list for a lung transplant.		PT	IC	Both elements of the dyad are strongly focused on the hope for a transplant in order to have QoL. The patient talks about her experience with the limitations imposed by the illness and how important the support of her family is. Her son talks about his experience when his mother has crises, and it is necessary to accompany her to the emergency department.	PT	40-48		PT	IC	In the second interview, their hope for a transplant is even more accentuated because the patient is moving forward in the process. Both are hoping that a donor will come as soon as possible and will be a suitable match.	Congruent The possibility that the patient receives a lung transplant is accentuated over time. The high HHI scores confirm these results.
		Factor 1 Temporal awareness	14	14		IC	44-48	Factor 1 Temporal awareness	16	16		
		Factor 2 Optimistic readiness	15	15				Factor 2 Optimistic readines	16	16		
		Factor 3 Interconnectedness	15	15				Factor 3 Interconnectedness	16	16		
F	Couple in which the husband is the patient (COPD) and the wife the caregiver. The patient has some limitations in daily living activities and so does the wife. This couple has no children, and their support network is limited.		PT	IC	The interview is very rich in emotions. Hope is associated with faith, religion, and health. Both members of the couple have health problems and have faith that one day they will be healed. The spouse regrets not having had children and talks a lot about loneliness, her eyes filling with tears when she talks about these issues. The patient demonstrates a great dependence on his wife for most of his activities.	PT	37-41		PT	IC	As in the first interview, in the second interview, hope and faith in God are intertwined; health and hope for better days are also still present in the couple’s experience. In this interview, themes such as faith and ways of demonstrating and cultivating faith are very much addressed. Current financial difficulties are also touched on during the interview, as well as the fact that they feel lonely.	Divergence In the second interview. Both elements of the dyad are hoping for better days; however, they describe their last months as difficult, which is incongruent with HHI scores.
		Factor 1 Temporal awareness	11	12		IC	38-41	Factor 1 Temporal awareness	13	13		
		Factor 2 Optimistic readiness	12	12				Factor 2 Optimistic readiness	14	14		
		Factor 3 Interconnectedness	14	14				Factor 3 Interconnectedness	14	14		
G	Couple in which the husband is the patient (pulmonary fibrosis) and the wife the main caregiver. The patient is in the process of waiting for a lung transplant. They are a very complicit couple.		PT	IC	Both members of the dyad focus on hope for a lung transplant. Both look forward to the arrival of the phone call that will change their lives and bring more QoL. The sources of hope for this couple revolve around faith, seeing their daughter grow up, and being in contact with nature.	PT	47-43		PT	IC	In the second interview, hope remains very much focused on the transplant. The patient says that his life is on pause waiting for this transplant that will change his life.	Congruent The transplant remains the focus of the dyad’s hope; however, it is described with less enthusiasm. HHI scores confirm these findings.
		Factor 1 Temporal awareness	16	15		IC	44-35	Factor 1 Temporal awareness	13	10		
		Factor 2 Optimistic readiness	15	15				Factor 2 Optimistic readiness	14	14		
		Factor 3 Interconnectedness	16	14				Factor 3 Interconnectedness	16	11		
H	The dyad is composed of two friends; the patient has ALS and the main caregiver is more than a person who receives a salary for the work she does. They are very complicit and are friends.		PT	IC	The patient focuses her hope on health (which she says she does not have), while the caregiver shows a positive attitude toward life where hope is always something positive. The patient in turn hopes that her symptoms will not worsen and that her situation will not deteriorate. Part of the patient’s family lives abroad, and she fears that if something happens, her children will not be able to see her.	PT	38-32		PT	IC	In the second interview, the patient says she has lost hope and is afraid of what might happen to her, but her family and friends help her through this difficult phase. Although saddened by the deteriorating situation, the patient says that the contact with nature helps to improve her state of mind. The caregiver maintains a positive attitude toward life and says that she has an inner strength that helps her to keep hope.	Congruent The experience of hope is described differently by the two members of the dyad. Patient and IC have different visions that are stable over time.
		Factor 1 Temporal awareness	9	15		IC	46-45	Factor 1 Temporal awareness	13	10		
		Factor 2 Optimistic readiness	14	16				Factor 2 Optimistic readiness	14	14		
		Factor 3 Interconnectedness	16	14				Factor 3 Interconnectedness	16	11		

Factor 1 - Inner sense of temporality and future (items 1, 2, 6, and 11) (range 4-16)

Factor 2 - Inner positive readiness and expectancy (items 4, 7, 10, and 12) (range 4-16)

Factor 3- Interconnectedness with self and others (items 3, 5, 8, and 9) (range 4-16)

*PT* Patient, *IC* Informal caregiver, *HHI* Herth Hope Index, *COPD* Chronic obstructive pulmonary disease, *QoL* Quality of life, *ALS* Amyotrophic lateral sclerosis

It presents a brief context of each dyad, as well as a description of both interviews at T1 and T2. Overall scores of hope of patients and informal caregivers are presented for T1 and T2 to enable comparison. The level of hope of dyads is presented in each factor of the HHI. In the last column of Table 6, we provide a comprehensive display of how both interviews and hope scores converge or diverge [55].

In our results, the data converge for all except Dyad F. At T2, Dyad’s F scores of hope were higher than at T1, which was not reflected in the interview of T2. Themes such as loneliness, financial difficulties, and loss of independence emerged extensively in the T2 interview. Both people in the dyad were hoping for better days, but during the interview, that was not the major topic of discussion; instead, they both held a plaintive attitude toward life.

## Discussion

This exploratory study is one of the first mixed-methods studies in a Portuguese context to explore the experience of hope as a changing dynamic over time for patients living with advanced chronic illness and their informal caregivers as a dyad, to evaluate hope and symptom burden, and to provide an expanded and more comprehensive understanding of the multidimensional phenomena of hope. Our findings suggest that hope is present in patients living with advanced chronic illness and their informal caregivers and that although its presentation may differ according to the trajectory of illness, it remains constant within their life transition. Even in the context of clinical deterioration and changing goals of care, hope was always present at some level in the dyads in this study, although hope transformed as needs and outcomes changed. This finding aligns with that of Buckley and Herth in their longitudinal study [58] and is important for nurses and other healthcare professionals who are in contact with patients transitioning toward palliative care. Nurses play a central role in recognizing, fostering, and supporting hope in patients with advanced chronic illness. Nurses are often at the forefront of providing care and support, and their understanding of the role of hope can significantly influence patients' experiences. Through open communication, active listening, and empathetic understanding, nurses can build trust with patients and informal caregivers. In practice, nurses can provide compassionate support by acknowledging and respecting dyads’ hopes, and they can educate patients and their families about the illness trajectory and help them set realistic goals based on their changing needs and circumstances [10]. In addition, nurses can ensure effective symptom management that enables patients to maintain their quality of life as much as possible in this challenging phase of their lives [59].

Although advanced chronic diseases and end of life care share common elements in terms of symptom management and psychological challenges, distinct elements of each need consideration [60]. Advanced chronic diseases are characterized by long-term management and may persist over years, sometimes even decades; the aim is to stabilize the disease, manage symptoms, and maximize functional ability to maintain quality of life [60]. In contrast, end of life care is typically provided in a short period, the focus being on providing comfort, dignity, symptom control, and psychological support, as well as facilitating a peaceful transition and maintaining quality of life [60, 61]. By recognizing and understanding the experience of hope of dyads living with advanced chronic diseases, healthcare professionals can better tailor interventions and support services to meet the unique needs of patients and their informal caregivers at different stages of the illness trajectory [13, 24, 40]. By providing palliative care, compassionate communication, holistic assessment, and person-centered care planning, nurses and other healthcare professionals can help individuals with advanced chronic illness and their informal caregivers navigate the complexities of living with chronic illness and transitioning to palliative care with dignity and support [13, 15].

The transition to palliative care in dyads living with advanced chronic illness is complex and it is difficult to determine the exact moment when it might occur. It is a rather slow movement in time determined by moments of crisis that occasionally define one moment of transition from one state to another. For dyads living with advanced chronic illness, the granularity of a transition cannot be associated only with change. People living with chronic illness experience physical deterioration over time, sometimes for decades, until the moment that physical and psychological symptoms take over their lives. The changes over time are sometimes unnoticeable such that people have time to adapt and bounce back. Transition as defined by Meleis [62] does not explain the granularity of the experience of dyads living with advanced chronic illness. The transition expressed in this study, more than passing from one state to another, is akin to going downstairs and having to adapt to each step that you descend; over time, the transition happens naturally but always toward decline.

The concept of transition in palliative care as a linear movement toward a specific outcome may be erroneous, given the fluidity of constantly changing health and risk of deterioration and death [63]. For some dyads, the experience of dealing with advanced chronic illness had been part of their daily life for a long time (for some, more than 20 years); the patients in these dyads may have established coping mechanisms and psychological defenses to deal with their condition. In this regard, the complexity of the transition towards palliative care, exhibited through negative emotions such as fear, anxiety, or a sense of giving up may not be fully represented by transition models that denote a movement to resolution. In this sense, transience, as a process of oscillation between permanence and impermanence may be a more useful descriptor to explain the experience of living with the consequences of advanced chronic diseases [63]. Nurses and other healthcare professionals need to navigate these emotions thoughtfully, ensuring that dyads feel supported and understood throughout the process.

Transitioning to palliative care may involve a shift in treatment goals from curative or disease management to symptom control and quality of life. For some patients, accepting this change may be challenging, and they may need guidance and support to align their care preferences with the goals of palliative care. Again, nurses play an essential role in facilitating discussions about goals of care, addressing concerns, and assisting patients in making informed decisions. Furthermore, transitioning to palliative care can be emotionally confronting for patients and informal caregivers, as it may involve acknowledging limitations and facing end of life issues. Nurses should provide emotional support, empathy, and counseling to help dyads navigate intricate emotional aspects of their situation. Nurses can also facilitate referrals to services such as specialized palliative care teams or psychotherapy, if needed. In addition, nurses need to be aware of the role of caregivers, as they may experience their own emotional and practical challenges. Nurses can provide guidance, education, and respite support to help alleviate the burden on informal caregivers and empower them to provide optimal care. Furthermore, the presence and engagement of an informal caregiver in the patient’s everyday life, as well as cultural beliefs and attitudes, shape how transitions are perceived [64].

At least four dyads referred to hope as something external to them (e.g., “hope for a lung transplant” or “hope for a donor”). This finding is interesting because the ways that healthcare professionals foster hope may shape the experience of dyads. It is fundamental for healthcare professionals to acknowledge the potential dangers of putting all patients' hopes in a single external factor, such as finding a donor. Although treatments or transplantation can bring hope, it is essential to maintain a balanced perspective and help patients and informal caregivers explore alternative sources of hope. By providing comprehensive support, including emotional and psychological support, healthcare professionals can better manage expectations and navigate the complex emotional challenges that arise when hope becomes dependent on external factors. Furthermore, through effective communication, active listening, and compassionate support, healthcare professionals can help dyads develop realistic expectations and goals while maintaining a sense of hope [65]. By providing accurate information about treatment options, prognosis, and potential alternatives, healthcare professionals can help patients and informal caregivers make informed decisions and explore hope beyond reliance on external factors alone.

The ESAS evaluation suggested that patients living with advanced chronic illness seemed to have their symptoms relatively controlled. This might be related to the fact that patients have stabilized their conditions over the years and are followed by a palliative care hospital-based team that provides them with support, management, and assessment of their symptoms. In addition, the collaborative nature of palliative care means that there is a multidisciplinary team of different healthcare professionals who contribute to effective symptom control and who facilitate comprehensive assessment, treatment planning, and ongoing monitoring of symptoms. Moreover, early palliative care interventions in patients with advanced chronic conditions could increase quality of life, improve symptom management, and motivate the writing of advance care directives [66].

### Study strengths and limitations

The strength of this study was the use of dyadic interviews. Although relatively unexplored as a research method, the dyadic interview was very much appreciated by all dyads. All participants were pleased that their loved ones could participate in the interview and share their points of view. Participants learned something from each other, and some difficult subjects came to the surface to be explored. The benefit of a dyadic interview over a single interview was in capturing the experience through the perspective of two persons who experience the consequences of advanced chronic illness together [50]. As a method, apart from the research interest, it has the potential to inform our understanding of shared experiences in complex situations and avoids the risk of compartmentalizing a situation. Instead, it situates the challenges in the real-world experience of the patient and informal caregiver and gives voice to both in expressing and determining their needs at critical transition points in living and dying [50]. In our study, dyads expressed their contentment in having the chance to share their experience of being accompanied by their loved ones. There were some moments of tension between interviewees; however, there were also opportunities for participants to deepen their shared experience. Despite the good feedback from participants, dyadic interview adds potential bias. One could imagine that the informal caregiver or the patient may not express their full experience because of the presence of the other.

The mixed-methods design allowed for extensive qualitative exploration, with findings strengthened by thorough triangulation. Nonetheless, there were also notable limitations.

The fact that the sample size is small meant that only descriptive statistical analysis was possible. Given the fragility of the population, the high risk of attrition posed an added challenge for research in this population [67]. Another limitation in this study was the difficulty in identifying changes between T1 and T2 because the time between the two moments was short. A much longer period would be required to study dyads experiencing advanced chronic illness. Evaluation of the quality of life of dyads would have also added more detailed information about the impact of symptoms on dyads’ everyday lives. Several informal caregivers mentioned the burden of caring for their loved ones. The evaluation of that burden quantitatively and qualitatively would have provided important information for healthcare professionals to design interventions that focus on relieving the informal caregiver burden.

Although the findings of this study provide valuable insights into the experiences of patients living with advanced chronic illness, as well as their informal caregiver, it is important to acknowledge that the small sample size means that the results should therefore be interpreted with caution, as they may not be fully generalizable to broader populations. However, it still enabled us to explore the appropriateness of conducting dyadic interviews in order to inform our future research.

### Implications for practice

Although a growing number of health professionals are recognizing the significance of hope and positive thinking in the context of patients with advanced chronic disease and their families, there remains a concern among other professionals that engaging in conversations regarding end of life care may diminish hope. There is a need for further training and practical experience in the realm of communication skills to assist healthcare professionals in striking a delicate balance between providing reliable and truthful information while also offering support to patients and their families as they navigate the process of adjusting to the inevitability of terminal illness. Although most hope-based interventions have been primarily targeted toward patients with cancer and their caregivers, there is evidence suggesting that some interventions may have positive effects in the context of many life-threatening illnesses.

Recognizing the multifaceted nature of hope and its different sources can help healthcare professionals provide holistic support to dyads. By acknowledging that hope can stem from various sources such as social support, spiritual beliefs, and personal connections, healthcare professionals can adopt a more comprehensive approach to supporting patients and their families. Encouraging dyads to explore and draw on these sources of hope can reinforce their resilience and emotional well-being, ultimately enhancing their coping abilities and quality of life.

## Conclusions

The findings of our study, which involved the dynamic analysis of dyads of patients with advanced chronic illness and their informal caregivers, show a persistent presence of hope even in the face of adversity. Hope is dynamic and multifaceted. Providing consistent symptom management is crucial to foster hope among dyads, which contributes to better quality of life and comfort for both patient and informal caregivers. Access to early palliative care in the context of chronic illness should be supported and promoted in order to follow up with patients and informal caregivers throughout their disease trajectory and to support hope for coping in challenging moments. Healthcare professionals should be continually updated and trained in communication skills to better respond to patients’ and informal caregivers’s expectations and avoid conveying messages of hope in a noxious way. Our study highlighted the importance of a shared experience within patient-caregiver dyads in which emotional support and open communication were central.

Advanced chronic diseases have an important impact on the lives of dyads that can persist over decades. This should be acknowledged by healthcare professionals and interventions should be designed to cover the needs of informal caregivers as well as those of patients. Healthcare systems benefit from the valuable service of informal caregivers in the care of advanced chronic patients, but the systems often fail to integrate informal caregivers in standard care. As our study shows, informal caregivers and patients may experience hope differently; however, by encouraging dyads to nurture hope, we reinforce their resilience and emotional well-being, ultimately enhancing their coping abilities and quality of life among the challenges posed by chronic illness.

Our study calls for continued investment in palliative care services that go beyond patient-focused symptom management and include dyads’ need for emotional and psychological support. More systemic interventions are needed to ensure a comprehensive and compassionate response to patients’ and informal caregivers’ needs. Further research is needed that includes dyads as a unit in order to better understand their dynamics and experiences.

### Supplementary Information

Supplementary Material 1.

Supplementary Material 2.

## Data Availability

The datasets generated during the current study are not publicly available because participants consented to participate but did not consent to sharing their data; however, these datasets are available from the corresponding author on reasonable request.

## Abbreviations

HHI: Herth Hope Index
ESAS: Edmonton Symptom Assessment System
COPD: Chronic obstructive pulmonary disease
ALS: Amyotrophic lateral sclerosis
CHF: Chronic heart failure
ILD: Interstitial lung disease
