# Supplementary material for: The experience of hope in dyads living with advanced chronic illness in Portugal: a longitudinal mixed-methods study
Source: BMC Palliat Care. 2024 Aug 14;23:207. doi: 10.1186/s12904-024-01528-x (PMC11325565; doi:10.1186/s12904-024-01528-x)
Supplement: Supplementary file 1 — Supplementary Material 1. [file 12904_2024_1528_MOESM1_ESM.docx]

Edmonton Symptom Assessment System

Bruera & al (1991) The Edmonton Symptom assessment system: A simple method for the assessment of palliative care patients.

Please circle the number that best describes your average symptom over the past 24 hours:

| No pain | 1 | 2 | 3 | 4 | 5 | 6 | 7 | 8 | 9 | 10 | Worst pain |
| --- | --- | --- | --- | --- | --- | --- | --- | --- | --- | --- | --- |
| No fatigue | 1 | 2 | 3 | 4 | 5 | 6 | 7 | 8 | 9 | 10 | Worst fatigue |
| No nausea | 1 | 2 | 3 | 4 | 5 | 6 | 7 | 8 | 9 | 10 | Worst nausea |
| No depressed | 1 | 2 | 3 | 4 | 5 | 6 | 7 | 8 | 9 | 10 | Worst depression |
| Not anxiety | 1 | 2 | 3 | 4 | 5 | 6 | 7 | 8 | 9 | 10 | Worst anxiety |
| No drowsiness | 1 | 2 | 3 | 4 | 5 | 6 | 7 | 8 | 9 | 10 | Worst drowsiness |
| No shortness of breath | 1 | 2 | 3 | 4 | 5 | 6 | 7 | 8 | 9 | 10 | Worst shortness of breath |
| Best appetite | 1 | 2 | 3 | 4 | 5 | 6 | 7 | 8 | 9 | 10 | Worst appetite |
| Best feeling or well being | 1 | 2 | 3 | 4 | 5 | 6 | 7 | 8 | 9 | 10 | Worst feeling of well being |
| Best sleep | 1 | 2 | 3 | 4 | 5 | 6 | 7 | 8 | 9 | 10 | Worst sleep |
