# Supplementary material for: The experience of hope in dyads living with advanced chronic illness in Portugal: a longitudinal mixed-methods study
Source: BMC Palliat Care. 2024 Aug 14;23:207. doi: 10.1186/s12904-024-01528-x (PMC11325565; doi:10.1186/s12904-024-01528-x)
Supplement: Supplementary file 2 — Supplementary Material 2. [file 12904_2024_1528_MOESM2_ESM.docx]

Herth Hope Index (HHI) – original version

Herth, K. (1992). Abbreviated instrument to measure hope: Development and psychometric

evaluation. Journal of Advanced Nursing, 17, 1251-1259.

| Listed below are a number of statements. Read each statement and place an [X] in the box that describes how much you agree with that statement right now. | | | | |
| --- | --- | --- | --- | --- |
|  | **Strongly**  **Disagree** | **Disagree** | **Agree** | **Strongly**  **Agree** |
| 1. I have a positive outlook toward life. |  |  |  |  |
| 2. I have short and/or long range goals. |  |  |  |  |
| 3. I feel all alone. |  |  |  |  |
| 4. I can see possibilities in the midst of difficulties. |  |  |  |  |
| 5. I have a faith that gives me comfort. |  |  |  |  |
| 6. I feel scared about my future. |  |  |  |  |
| 7. I can recall happy/joyful times. |  |  |  |  |
| 8. I have deep inner strength. |  |  |  |  |
| 9. I am able to give and receive caring/love. |  |  |  |  |
| 10. I have a sense of direction. |  |  |  |  |
| 11. I believe that each day has potential. |  |  |  |  |
| 12. I feel my life has value and worth. |  |  |  |  |

The scoring consists on the sum of the points for the subscale and for the total scale.

Subscales are based on the three factors :

- Factor 1 – inner sense of temporality and future
- Factor 2 - inner positive readiness and expectancy
- Factor 3 – interconnectedness with self and others

(see Table 2 in 1992 publication)

Total possible points on the total scale is 48 points. The higher the score the higher the level of hope.

The items 3, 6 need to be reversed. Score items as follows:

Strongly Disagree = 1

Disagree = 2

Agree = 3

Strongly Agree = 4
